# *E6* and *E7* gene polymorphisms in human papillomavirus Type-6 identified in Southwest China

**DOI:** 10.1186/s12985-019-1221-x

**Published:** 2019-09-12

**Authors:** Zuyi Chen, Qiongyao Li, Jian Huang, Jin Li, Feng Yang, Xun Min, Zehui Chen

**Affiliations:** 1 Department of Laboratory medicine, Affiliated Hospital of Zunyi Medical University, Zunyi, Guizhou People’s Republic of China; 2 Bio-resource Research and Utilization Joint Key Laboratory of Sichuan and Chongqing, Chongqing, Sichuan People’s Republic of China; 3 Department of Information Technology, Affiliated Hospital of Zunyi Medical University, Zunyi, Guizhou People’s Republic of China; 4 Department of Thoracic Surgery, Affiliated Hospital of Zunyi Medical University, Zunyi, Guizhou People’s Republic of China

**Keywords:** HPV6, Genetic diversity, E6, E7, Positive selection

## Abstract

**Background:**

Human papillomavirus type-6 (HPV6) is the major etiological agent of anogenital warts both men and women. The present study aimed to characterize the genetic diversity among HPV6 in Southwest China, and to investigate the origin of, selective pressure experienced by, and impact of the resultantly identified genetic variants on the HPV6 secondary structure.

**Methods:**

Phylogenetic trees were constructed by Maximum-likelihood and the Kimura 2-parameters methods by Molecular Evolutionary Genetics Analysis version 6.0. The diversity of secondary structure was analyzed by PSIPred software. The selection pressures acting on the *E6*/*E7* genes were estimated by Phylogenetic Analyses by Maximum Likelihood version 4.8 software.

**Results:**

HPV6 was the most prevalent low risk HPV type in southwest China. In total, 143 *E6* and *E7* gene sequences of HPV6 isolated from patients were sequenced and compared to GenBank HPV6 reference sequence X00203. The results of these analyses revealed that both the HPV6 *E6* and *E7* were highly conserved within the analyzed patient samples, and comprised only 3 types of variant sequence, respectively. Furthermore, the analysis of HPV6 *E6* and *E7* sequences revealed seven/five single-nucleotide mutations, two/four and five/one of which were non-synonymous and synonymous, respectively. The phylogenetic analyses of the *E6* and *E7* sequences indicated that they belonged to sub-lineage A1 and sub-lineage B1, whereas the selective pressure analyses showed that only the *E7* mutation sites 4R, 34E, and 52F were positive selection.

**Conclusions:**

HPV6 (detection rate = 13.10%) was very prevalent in southwest China, both the HPV6 *E6* and *E7* sequences were highly conserved within the analyzed patient samples in southwest China, indicating that the low risk HPV6 can adapt to the environment well without much evolution.

## Introduction

Human papillomaviruses (HPVs) are associated with a variety of epithelial lesions, including benign genital warts and cervical intraepithelial neoplasia [1]. To date, more than 250 HPV types have been identified and each of these genotypes are associated with infection at particular anatomical sites. HPV6 may be the most prevalent low risk alpha-papillomavirus type and is commonly associated with genital warts [2]. For example, anogenital warts are primarily caused by HPV6 (family *Papillomaviridae*, genus *Alphapapillomavirus*, species 10) [3], which brings a significant burden to both the healthcare system and patients. Similarly, one third of Dutch primary school children have cutaneous warts, of which approximately 20% seek medical treatment each year [4]. Generally speaking, these infections are classified as “not carcinogenic” or “low risk”, they often attract negative attention, and thereby cause significant psychological distress [5]. However, some HPV6 variants are classified as “carcinogenic”, because they cause infections that lead to potentially fatal conditions, such as tonsillar, and malignant laryngeal carcinoma and/or malignant laryngeal papilloma [6–9].

To date, extensive research has been conducted to investigate sequence variation among carcinogenic HPV types; nevertheless, only limited data is available regarding HPV6 variants, despite their significant impact on human health. Structurally, the HPV is a double-stranded, circular DNA virus that encodes E1, E2, E4, E5, E6, E7, L1 and L2 proteins [10]. HPVs infect cells via the basal layer of the stratified epithelium, and viral gene expression is closely linked to the endogenous differentiation program of the host cells [11]. Of the HPV-encoded proteins, E6 and E7 have been shown to be the most important pathogenic HPV proteins. They have been previously shown to function as oncoproteins that critically regulate HPV-induced tumorigenesis [12]. Furthermore, they have also been demonstrated to be essential to maintain the extrachromosomal forms of HPV in undifferentiated basal cells [13].

Genetic variability analyses have proven essential to facilitate an improved understanding of the evolution of the papillomavirus. A number of carcinogenic variants have been identified in HPV variants isolated from populations in Southwest China; however, only limited research has been conducted to identify low risk HPV variants. Thus, the present study aimed to analyze *E6* and *E7* sequence variability among HPV6 isolated from cervical papilloma samples collected from patients in Southwest China. Phylogenetic analyses were conducted to compare the identified nucleotide sequences with those previously described in other ethnic populations. In addition, the secondary structure of the identified sequences were predicted to assess the probably impact of the low risk variants on overall viral function. The results of the study could provide important data for the research on HPV6 prevention, diagnostic, therapeutic and even the design of therapeutic vaccines based on proteins E6 and E7 in Southwest China.

## Methods & Materials

### Clinical samples and HPV typing

From May 8, 2013 to June 1, 2016, cervical swabs were obtained from patients (with informed consent and ethical approval) at the Affiliated Hospital of Zunyi Medical University, Angel Women’s and Children’s Hospital, Sichuan Reproductive Health Research Centre Affiliated Hospital, and the Chengdu Western Hospital Maternity Unit. Women over 18 years old with visible cervical lesions and/or HPV-related diseases (e.g. cervical papilloma) were eligible for inclusion. Specimens were stored at − 20 °C until DNA extraction and HPV typing. Specimens DNA were extracted and tested using the Human Papillomavirus Genotyping Kit For 23 Types (PCR-RDB, reverse Dot Blot) according to the manufacturer’s instructions (Yaneng Bio, Shenzhen, China). This kit enabled the classification of the 23 HPV types (HPV 16, 18, 31, 33, 35, 39, 45, 51, 52, 53, 56, 58, 59, 66, 68, 73, 83, MM4, 6, 11, 42, 43, and 44).

### PCR amplification

In total, 216 samples were subjected to PCR amplification with *E6* and *E7* gene primers (see Additional file 1) that were designed using Primer Premier 5.0 software (Premier Biosoft, California, USA) and the HPV6 reference sequences (X00203) listed in the GenBank database (https://www.ncbi.nlm.nih.gov/genbank/). The reaction mixture comprised 2.5 mM dNTPs, 2 U Taq DNA polymerase, 90 pmol of each primer, and sufficient 10× Mg^2+^ PCR buffer to reach a final volume of 25 μL. Amplification of the sample sequences was achieved using thermal cycling conditions comprising an initial denaturation step (5 min at 95 °C), followed by 39 amplification cycles (each consisting of a 45 s denaturation step at 94 °C, a 60 s annealing step at 53 °C, and a 60 s elongation step at 72 °C). All amplified HPV6 *E6* and *E7* DNA products were detected via electrophoresis using an agarose gel that was impregnated with ethidium bromide, and sequenced using Sanger sequencing (Sango Biotech, Shanghai, China), and all the data were confirmed by repeating the PCR amplification and sequence analysis at least twice.

### Sequence analysis

The secondary structures of the identified sequences were predicted with PSIPRED online software v.3.2 (http://bioinf.cs.ucl.ac.uk/psipred/), using default parameters. This software uses a very stringent cross-validation technique to achieve an average Q3 score of 81.6% [14]. Any mutation observed to occur with a frequency of ≥10% was considered to be a major mutation. The sequences and variants were subsequently analyzed with various other software programs, including NCBI BLAST (https://blast.ncbi.nlm.nih.gov/Blast.cgi), Primer Premier 5 (Premier Biosoft), and DNAMAN version 5.2.2 (Lynnon Biosoft, California, USA). HPV6 nucleotide positions were numbered in accordance with the GenBank HPV6 reference sequence (X00203).

### Phylogenetic analysis of the identified HPV6 variants

“Maximum-likelihood” phylogenetic trees were constructed for the identified *E6* and *E7* variants using MEGA v.6 software (http://www.megasoftware.net/home) to apply Kimura’s two-parameter model. Tree topology was evaluated by bootstrap resampling 1000 times [15]. The reference viral sequences used to construct the distinct phylogenetic branches were collected from the GenBank database. Numbers above the branches indicate the bootstrap values that are greater than 60%.

### Selective pressure analysis

Sites within the *E6* and *E7* HPV6 gene sequences that were likely to be subject to positive selection were predicted by using PAML 4.8 software (codeml tool; http://abacus.gene.ucl.ac.uk/software/paml.html) to perform likelihood ratio tests (LRTs) to infer nonsynonymous/synonymous nucleotide divergence, according to the method described by Nei and Gojobor [16–18]. The E6 and E7 protein sequences were aligned using ClustalX v. 2.1 software (ftp://ftp.ebi.ac.uk/pub/software/clustalw2/) [19].

### Cross-sectional study

The sequence results were compared to reference sequences in NCBI (https://www.ncbi.nlm.nih.gov/nuccore/). Seven reference sequences were obtained (KU298876; HE599232; FR751328; FR751337; AF092932; L41216; JN252318).

## Results

### Characteristics of HPV6 prevalence in Southwest China

**Fig. 1:**
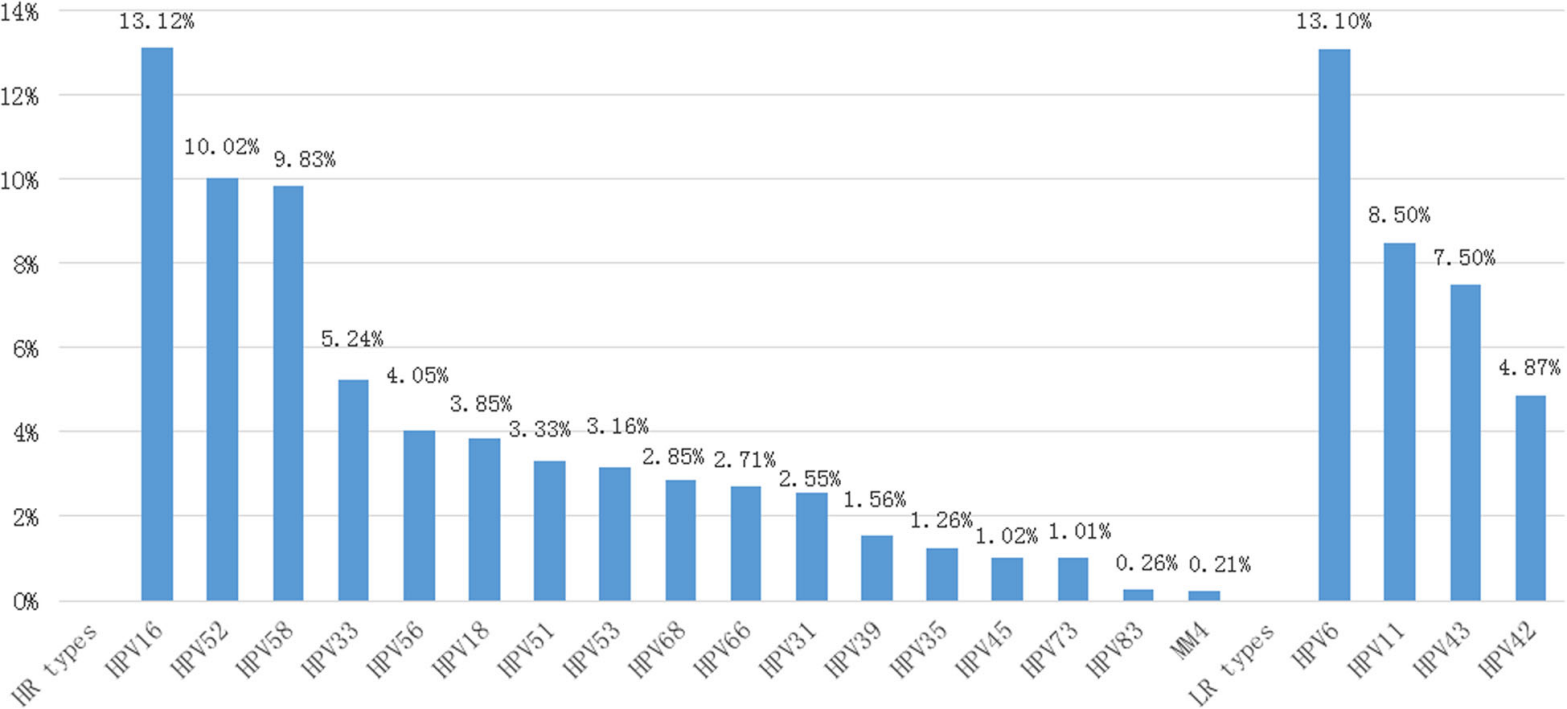
Detection result of 23 human papillomavirus types among 9349 women. Note: Ordinate indicates detection rate, abscissa represents the different HPV types detected. HR indicates high-risk, LR indicates low-risk

Nine thousand, three hundred and forty nine specimens (mean age 31.56 ± 8.42 years old) were collected. The overall positive rate of detectable 23 HPV types was 33.33% (3116/9349). 22.01% (2058/9349) specimens were high risk HPV types and 11.32% (1058/9349) were low risk HPV types. In all HPV-positive women involved in the study, the most common detected HPV types were HPV16 (detection rate = 13.12%) and HPV6 (detection rate = 13.10%) (Fig. 1). HPV6 was the most prevalent low risk HPV, followed by HPV11 (265, 8.50%), HPV-43 (234, 7.50%) and HPV42 (152, 4.87%). No HPV59 and HPV44 types were detected in the patients tested.

### *E6* sequence variations

**Fig. 2:**
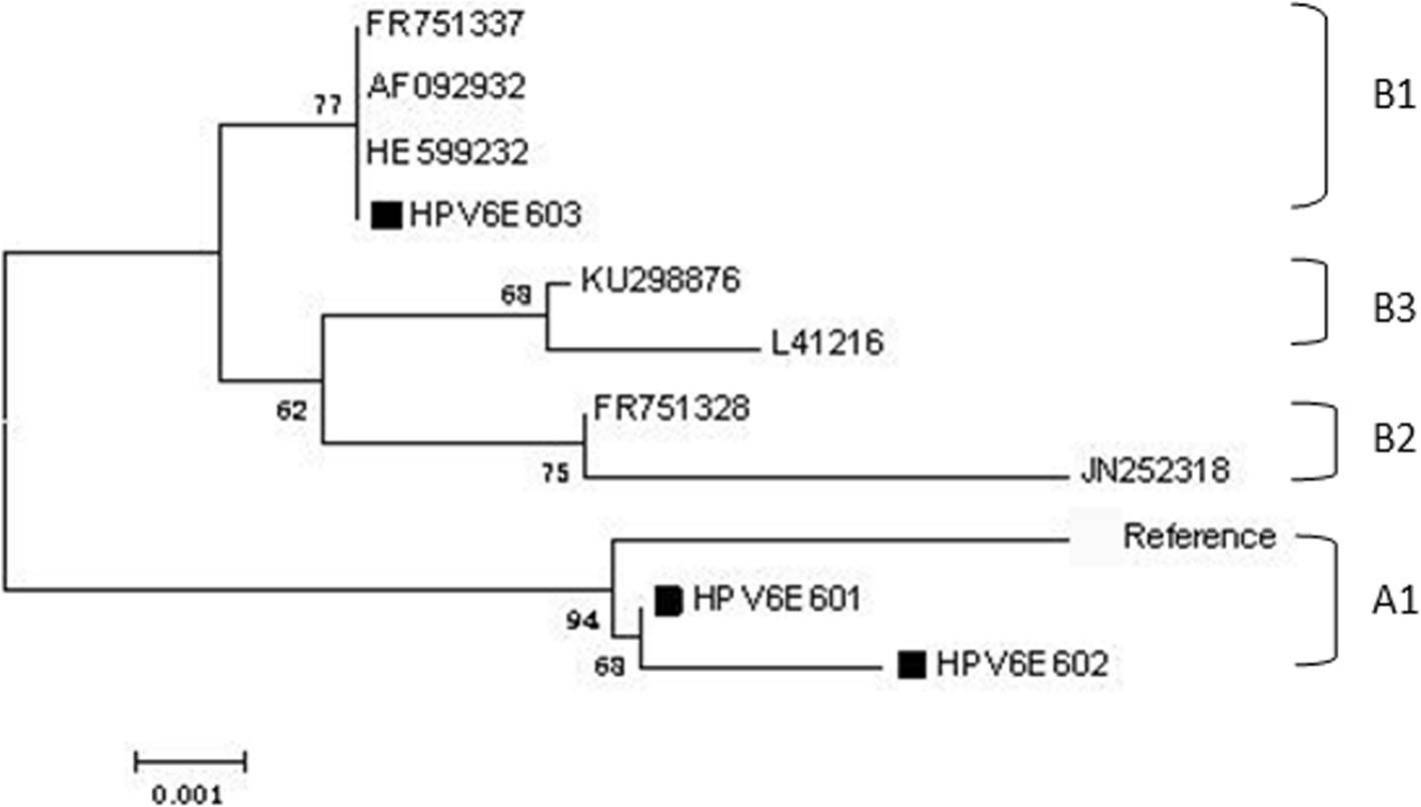
The Maximum-likelihood tree of HPV6 *E6* variants based on combined sequences. Note: Numbers above the branches indicate the bootstrap values that are greater than 60%. The sample sequences are labeled in black square, others are standard sequences

In total, 143 HPV6 *E6* gene sequences were obtained from the analyzed patient specimens using Sanger sequencing. A comparison of these with the GenBank HPV6 reference sequence (X00203) identified seven polymorphic sites, two of which comprised non-synonymous (D14Y, H50Q), and five of which comprised synonymous mutations. One of the identified non-synonymous mutations, D14Y, occurred within a region that encodes the alpha helix, whereas three of the synonymous mutations occurred in regions encoding the α-helix or β-strand. Notably, the mutation A120T and G372A were discovered in all specimens (Table 1). The table is designed with the reference to previous study [20, 21]. No insertion or deletion mutations, nor any evidence of introduced premature stop codons, were detected within the analyzed *E6* HPV6 sequences. The conducted alignment of the analyzed (453 bp) *E6* sequences with reference sequences belonged to sub-lineage A1 (70.63%) and sub-lineage B1 (29.37%) (Fig. 2).

**Table 1:** Nucleotide sequence mutation at *E6* of HPV6 isolates

Sequence pattern	HPV6 *E6*							Number of samples	Sub-lineages
	40	120	150	222	264	291	372		
Reference	G	A	C	A	A	C	G		
HPV6E601	.	T	.	.	.	.	A	96	A1
HPV6E602	T	T	.	.	.	.	A	5	A1
HPV6E603	.	T	G	T	T	T	A	42	B1
Reference AA	D	T	H	I	T	I	A		
AA position	14	40	50	74	88	97	124		
AA mutations	Y	.	Q	.	.	.	.		
Secondary structure	H		H	H		S			

Note: The nucleotides conserved with respect to the reference sequence were marked with a dash (.), whereas a variation position was indicated by a letter. The “S” in the last row of the table means Strand, the “H” means Helix. The “AA” means amino acid. The “D, T, H, I, A, Y, Q” in the seventh and ninth rows of the table represent aspartic acid, threonine, histidine, isoleucine, alanine, tyrosine, and glutanine, respectively. The numbers in the second row of the table mean nucleotide positions

### *E7* sequence variations

**Fig. 3:**
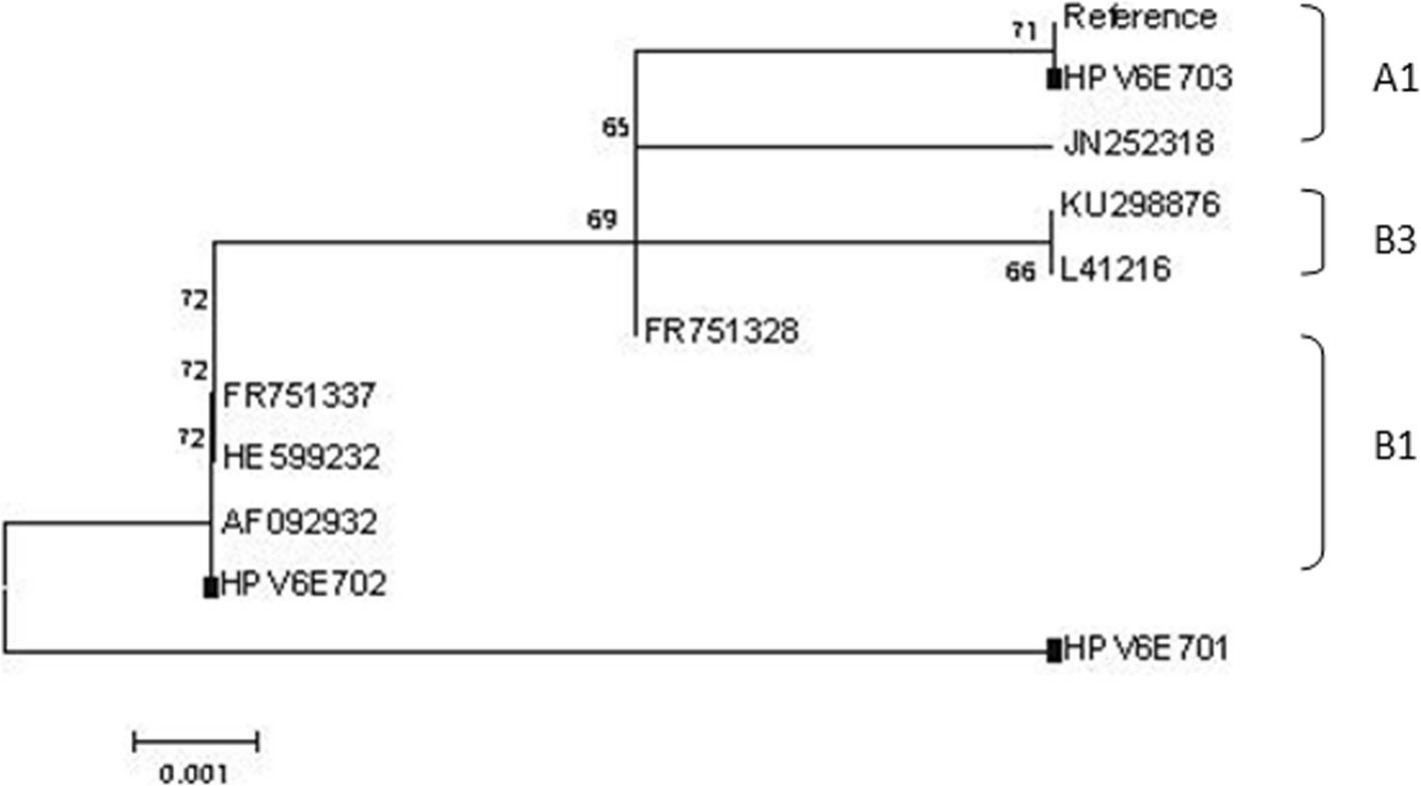
The Maximum-likelihood tree of HPV6 *E7* variants based on combined sequences. Note: Numbers above the branches indicate the bootstrap values that are greater than 60%. The sample sequences are labeled in black square, and others are standard sequences

Compared with the HPV6 reference sequence (X00203), five single-nucleotide changes were identified within the 297 bp *E7* open reading frame (ORF), of which four substitutions were non-synonymous, and one substitutions were synonymous. The most common non-synonymous mutation, T155A, occurred with a frequency of 34.27%, resulted in the amino acid (AA) change of F52Y (Phe to Tyr). The C294A was the most common synonymous mutation, and occurred with a frequency of 34.26%. One non-synonymous mutation (F52Y) occurred in the *E7* sequence encoding β-strand (Table 2). No insertions, deletions, or premature stop codons were identified within the analyzed *E7* variants. The (297 bp) *E7* variants mainly belonged to sublineages A1 (65.73%) and sublineages B1 (31.47%) (Fig. 3). HPV6E701 was newly found.

**Table 2:** Nucleotide sequence mutation at E7 of HPV6 isolates

Sequence pattern	HPV6 E7					Number of samples	Sub-lineages
	10	11	100	155	294		
Reference	A	G	G	T	C		
HPV6E701	G	A	A	A	A	4	
HPV6E702	.	.	.	A	A	45	B1
HPV6E703	.	.	.	.	.	94	A1
Reference AA	R		E	F	T		
AA position	4		34	52	98		
AA mutations	E		K	Y	.		
Secondary structure				S			

Note: the nucleotides conserved with respect to the reference sequence were marked with a dash (.), whereas a variation position was indicated by a letter. The “S” in the last row of the table means Strand, the “H” means Helix. The “AA” means amino acid. The “R, E, F, T, K, Y” in the seventh and ninth rows of the table represent arginine, glutamic acid, phenylalanine, threonine, lysine and tyrosine, respectively. The numbers in the second row of the table mean nucleotide positions

### Structural analysis

None of the identified variant sequences were predicted to introduce changes to the reference HPV6 E6 and E7 secondary structures (see Additional files 2, 3, 4 and 5). Overall, the secondary structure of the E6 protein was predicted to consist of 30.0% helix, 16.7% strand, and 53.3% coil elements, whereas that of the E7 protein was predicted to comprise 12.3% helix, 18.4% strand, and 69.3% coil elements. There was no obvious change in the predicted HPV6 E6 and E7 secondary structures.

The non-synonymous/synonymous rate ratio (dN/dS), can measures selective pressure. When one non-synonymous mutation has advantage in fitness, it will rise to a higher rate than synonymous mutation, resulting in dN/dS > 1. The variable dN/dS ratios were tested among various lineages using the PAML 4.8 software. This program uses the Nei and Gojobori method of estimating synonymous substitutions, which is an unweighted pathway method. The first step in the procedure requires enumeration of the number of synonymous and non-synonymous sites present at each codon, where each site may be both partially synonymous and non-synonymous. The next stage is to determine the number of synonymous and non-synonymous changes between each pair of aligned sequences, codon-by-codon. The results of the conducted selective pressure (i.e, variable dN/dS ratio) analysis of the identified HPV6 *E6* and *E7* gene sequences are summarized in Tables 3 and 4. No positively selected sites were identified within the *E6* sequence; however, in contrast, the *E7* variant sites R4E (arginine to glutamic acid), E34K (glutamic acid to lysine), and F52Y (phenylalanine to tyrosine), were predicted to be subjected to positive selection.

**Table 3:** Site-specific tests for positive selection on HPV6 E6

Models	InL	Estimates of parameters	2Δl	Positively selected sites
M7	− 662.94	*p* = 12.39037 q = 99.00000		NA
M8	−662.94	p0 = 0.99999 *p* = 12.38952 q = 99.00000 (p1 = 0.00001) w = 1.00000	0 P = 1	NONE

Note: lnL, the log-likelihood difference between the two models. 2∆l, twice the log-likelihood difference between the two models (*P* < 0.05 was considered statistically significant). NA means not applicable. M7 means specifies the model of nucleotide substitution (REV); M8 means specifies the model of nucleotide substitution (UNREST)

**Table 4:** Site-specific tests for positive selection on HPV6 E7

Models	InL	Estimates of parameters	2Δl	Positively selected sites
M7	− 436.09	*p* = 1.36732 q = 0.00500		NA
M8	− 433.89	p0 = 0.93931 *p* = 0.00500 q = 8.82347 (p1 = 0.06069) w = 29.56283	4.40 *P* < 0.01	4R, 34E, 52F

Note: lnL, the log-likelihood difference between the two models. 2∆l, twice the log-likelihood difference between the two models (P < 0.05 was considered statistically significant); NA means not applicable. M7 means specifies the model of nucleotide substitution (REV); M8 means specifies the model of nucleotide substitution (UNREST)

### The analysis of sequence results and reference sequence

Comparation between the results of our sequence with the reference sequence has been listed in the Table 5. For *E6*, the mutations A120T, C150G, A264T, and C291T were discovered in all sequence listed. The mutation G40 T was only found in HPV6vc (Table 5). Point mutations altered the *E6* AA sequence of non-prototypic isolates, which contained specific mutations at nucleotide positions 150 (H50Q). AA exchanges identified at nucleotide positions 40 (D14Y) were additional characteristic features. The mutation at nucleotide positions 55 (T19S) was not found in this study. For E7, A10G, G11A, and G100A were proved as new mutations in this study. The mutations C156T and A262G were not discovered in the research. All sequence listed contain the mutation C294A. Point mutations altered the E7 AA sequence of non-prototypic isolates, which contained specific mutations at nucleotide positions 155 (F52K). AA exchanges identified at nucleotide positions 10, 11 (R4E) and 100(E34K) were additional characteristic features. The mutation at nucleotide positions 262 (D88N) was not found in this study.

**Table 5:** Sequence results compared to reference sequences

Sequence	HPV6 E6											HPV6 E7						
	40	55	120	150	159	222	264	279	291	372	378	10	11	100	155	156	262	294
Reference	G	A	A	C	C	A	A	T	C	G	C	A	G	G	T	C	A	C
HPV6vc	T	.	T	G	.	T	T	.	T	A	.	G	A	A	A	.	.	A
KU298876	.	.	T	G	.	C	T	.	T	A	T	.	.	.	.	.	G	A
HE599232	.	.	T	G	.	T	T	.	T	A	.	.	.	.	A	.	.	A
FR751328	.	T	T	G	.	C	T	.	T	A	.	.	.	.	.	.	.	A
FR751337	.	.	T	G	.	T	T	.	T	A	.	.	.	.	A	.	.	A
AF092932	.	.	T	G	.	T	T	.	T	A	.	.	.	.	A	.	.	A
L41216	.	.	T	G	.	C	T	.	T	.	T	.	.	.	.	.	G	A
JN252318	.	T	T	G	T	C	T	G	T	A	.	.	.	.	.	T	.	A
Reference AA	D	T		H								R		E	F		D	
AA position	14	19		50								4		34	52		88	
AA mutations	Y	S		Q								E		K	K		N	

Note: The nucleotides conserved with respect to the reference sequence were marked with a dash (.), whereas a variation position was indicated by a letter. The numbers in the second row of the table mean nucleotide positions. “HPV6vc” includes all mutations of the sequenced samples. AA means amino acid

## Discussion

E6 and E7 are essential HPV E-gene products, they target P53 and retinoblastoma (Rb) tumuor-suppressor proteins, respectively. The degradation of PRb can initiate abnormal cell replication; the inhibition of *p53* can cause abnormal replication cell to lose control. The HPV genome can only be replicated along with the replication of the host genome. Therefore, *E6* and *E7* are especially important in HPV lifecycle. The present study sequenced the *E6* and *E7* ORFs in 143 HPV6 isolates from patients in Southwest China, to assess the HPV6 genetic diversity and evolution characteristics within this population, and help further identify specific HPV6 variants.

HPV6 was the second prevalent HPV type and the most prevalent low risk HPV type in southwest China, indicating that HPV6 is highly adaptable to the environment in southwest China compare with most HPV types that are not so prevalent.

Seven *E6* sequence variants were identified, two of which induced corresponding *E6* amino acid changes. One of these, H50Q (His to Gln), was identified in 30.08% of the analyzed HPV6 sequences, the amino acid at position 50 of prototype HPV6 E6 may be located centrally between the two internal Zn-binding motifs, which is important for the E6 protein stability [22]. These variants have not been discovered previously in Southwest China, and their functional impact requires further analysis. Overall, the HPV6 *E6* sequence was shown to be highly conserved within the analyzed patient samples, which exhibited only two main genotypes, HPV6E601 and HPV6E603, that represented 67.13% (96/143) and 29.37% (42/143) of the samples, respectively. Likewise, five *E7* variants were identified, four of which exhibited a corresponding *E7* amino-acid change. The most common *E7* nucleotide mutations were T155A and C294A. Notably, T155A mutation affected 34.27% (49/143) patients, and caused a F52Y (Phe to Tyr) amino acid conversion that was predicted impact the protein’s secondary structure. Furthermore, the *E7* sequence also was proved to be highly conserved within the patient specimens, comprising only two main genotypes HPV6E702 and HPV6E703 that were exhibited by 31.47% (45/143) and 65.73% (94/143) of the analyzed patients, respectively, and the sequence of HPV6E703 is in conformity with the reference (X00203). The fact that both sequences were so highly conserved supports the hypothesis that they play vital roles in HPV6 structure and function [23, 24], consistent with previous studies by Dartmann [25]. Thus, they are likely also promising targets for HPV6 primer design and diagnostic detection. Compared to deadly cancerogenic high risk HPV, low risk HPV is able to get along better with the host; low risk HPV is more likely to be ignored in clinical treatment and prevention. HPV6 *E6* and *E7* were much more conserved than main high risk HPV types (like 16, 33, 53 and 58), may indicating low risk HPV has lower evolutionary pressure in gene level [20, 21, 26].

Structurally, some of the detected mutations affected amino acids at critical positions related to known biological functions. In E6, one (I97) and three (D14Y, H50Q, and I74) mutations were found to affect the β-strand and α-helix-encoding regions, respectively, that are critical for structural stability. In E7, only one mutation (F52Y) was detected may affect the β-strand-encoding region. The nucleotides as positions 58, 61, 91, and 94 in the E7 protein sequence have been previously shown to act as zinc binding sites [27], but no mutations were identified by the present study to occur in these positions, nor in the consensus LXCXE RB1-binding site (positions 22–26), or zinc-binding motif.

Notably, the present study is first to conduct an analysis to assess whether the *E6* and *E7* sequences are subject to positive selection in Southwest China. The main characteristic of positive selection is that it causes an unusually rapid rise in allele frequency, thereby enabling a species to adapt rapidly to environmental changes [28]. The selective pressure analysis showed that all the sites that evolved under positive selection were common non-synonymous mutations, indicating that the positively selected variations beneficial for HPV6 to accommodate their environments are wide-spread. Moreover, remarkably, the positive sites R4E, E34K, and F52Y were observed in HPV6 *E7*, they may have evolutionary significance in making HPV6 adaptive to their environments.

Previous studies revealed the existence of two variant lineages (lineage A, lineage B) and five variant sub-lineages (sub-lineage B1, B2, B3, B4, B5) [29] among HPV6 variants. The conducted phylogenetic analyses showed that 97.56% of the *E6* and *E7* sequences within the patient specimens belonged to sub-lineage A1 (Reference, HPV6b) and sub-lineage B1 (HE599232, LP243) [29]. Lineage A has been shown to predominate in Asia; in contrast, lineage B is distributed globally [30, 31], and consists mainly of variants from sub-lineage B1. Given its global distribution, lineage B1 may represent the oldest HPV6 sub-lineage, and likely disseminated during early human evolution. This may have enabled it to migrate to different regions of the world prior to the emergence of other HPV6 sub-lineages [29], and for *E7,* a new sub-lineage was found, however, only three samples were detected, and therefore, it requires further study.

Certain strengths of this study were that all clinical samples were collected from the Southwest region of China which had a strong regional representation and several new mutations were discovered, which will provide real and valid data for development of therapeutic vaccines for affected people in the Southwest China. Thus, the results of the present study significantly expand the current knowledge of HPV6 genetic diversity in Southwest China, and also provide a valuable resource for future studies of HPV6 epidemiology, evolution, function, pathogenesis, and use as a therapeutic target. However, the cellular level research is required, and a series of cellular experiments about the mutations should be designed for future studies.

## Conclusions

HPV6 was very prevalent in southwest China, both the HPV6 *E6* and *E7* sequences were highly conserved within the analysed patient samples in southwest China, may indicating that the low risk HPV6 can adapt to the environment well without much evolution.

## Supplementary information

**Additional file 1: Table S1.** HPV6 *E6* and *E7* primers. (DOC 32 kb)

**Additional file 2:** HPV6 E6 reference sequence predicted secondary structure. Secondary structure within the reference sequence of HPV6 E6 protein. (PDF 14 kb)

**Additional file 3:** HPV6 E6 variation sequence predicted secondary structure. Secondary structure within the variation sequence of HPV6 E6 protein. Variations of HPV6E601, HPV6E602 and HPV6E603 were integrated into one sequence to predict. (PDF 15 kb)

**Additional file 4:** HPV6 E7 reference sequence predicted secondary structure. Secondary structure within the reference sequence of HPV6 E7 protein. (PDF 11 kb)

**Additional file 5:** HPV6 E7 variation sequence predicted secondary structure. Secondary structure within the variation sequence of HPV6 E7 protein. Variations of HPV6E701, HPV6E702 and HPV6E703 were integrated into one sequence to predict. (PDF 11 kb)

## Data Availability

All data generated or analyzed during this study are included in this published article and GeneBank.

## Abbreviations

AA: Amino acid
BEB: Bayes empirical Bayes
HPV: Human papillomavirus
MEGA: Molecular evolutionary genetics analysis version
ORF: Open reading frame
PAML: Phylogenetic analyses by maximun likelihood
Rb: Retinoblastoma
