# Supplementary material for: E6 and E7 gene polymorphisms in human papillomavirus Type-6 identified in Southwest China
Source: Virol J. 2019 Sep 12;16:114. doi: 10.1186/s12985-019-1221-x (PMC6740006; doi:10.1186/s12985-019-1221-x)
Supplement: Supplementary file 1 — Additional file 1: Table S1. HPV6 E6 and E7 primers. (DOC 32 kb) [file 12985_2019_1221_MOESM1_ESM.doc]

*E6* and *E7* Gene Polymorphisms in Human Papillomavirus Types-6 Identified in Southwest China

Zuyi Chen^ad¶^, Qiongyao Li^bd¶^, Jian Huang^a^, Jin Li^c^, Feng Yang^b^, Xun Min^a^*, Zehui Chen

**Supplementary Table 1. HPV6 *E6* and *E7* primers**

| Primer name | Primer sequence | product length | Annealing temperature |
| --- | --- | --- | --- |
| HPV-6 *E6* F | 5ʹ TTTTGCTCTTACTGTTTGG 3ʹ | 453bp | 53°C |
| HPV-6 *E6* R | 5ʹ AGGGTCTGGAGGTTGC 3ʹ | 453bp | 53°C |
| HPV-6 *E7* F | 5ʹ GGTCGCTGCCTACACT 3ʹ | 297bp | 53°C |
| HPV-6 *E7* R | 5ʹ CGTCCGCCATCGTTA 3ʹ | 297bp | 53°C |

Note: F, forward primer; R, reverse primer.
