# Supplementary figures and images for: E6 and E7 gene polymorphisms in human papillomavirus Type-6 identified in Southwest China
Source: Virol J. 2019 Sep 12;16:114. doi: 10.1186/s12985-019-1221-x (PMC6740006; doi:10.1186/s12985-019-1221-x)

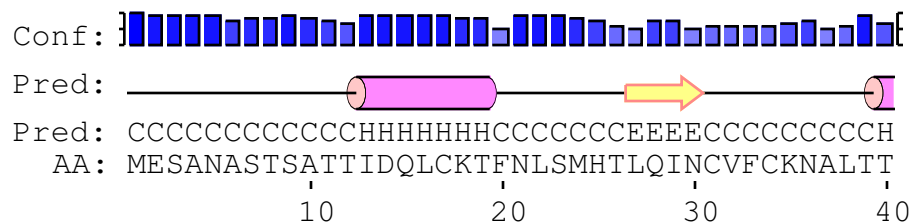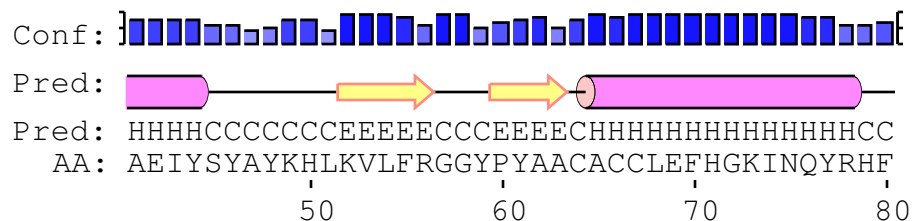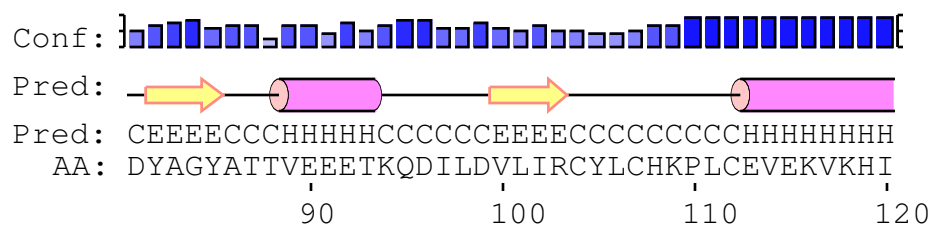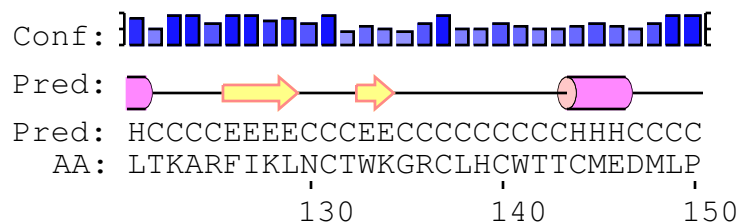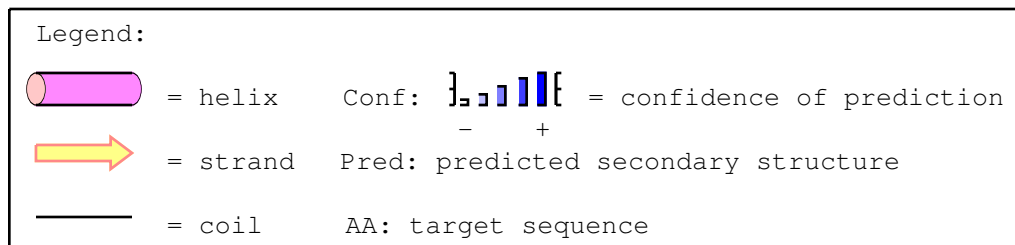

Supplement: Supplementary file 2 — Additional file 2: HPV6 E6 reference sequence predicted secondary structure. Secondary structure within the reference sequence of HPV6 E6 protein. (PDF 14 kb) [file 12985_2019_1221_MOESM2_ESM.pdf]

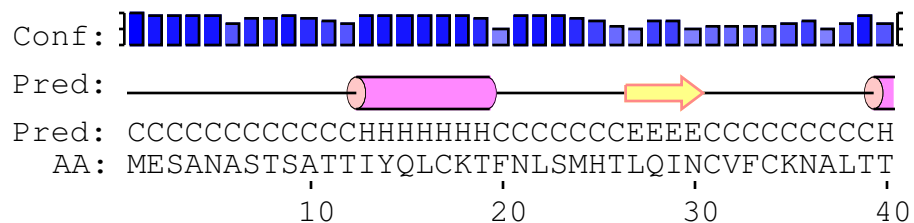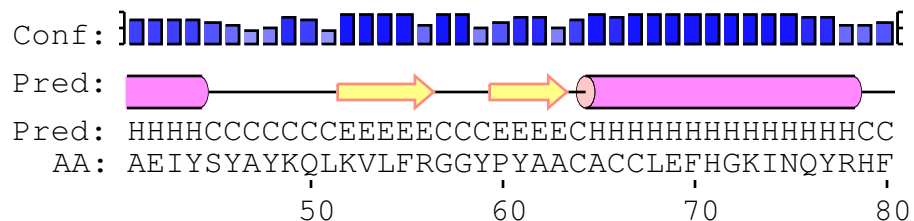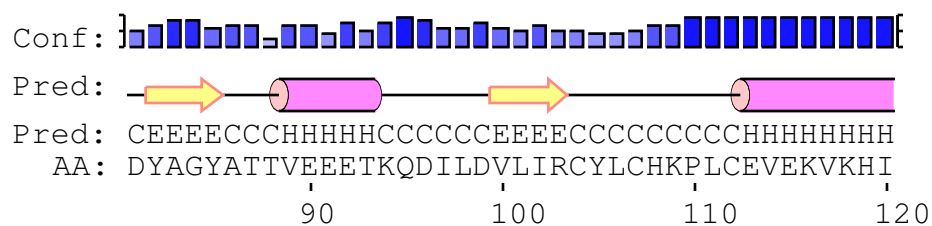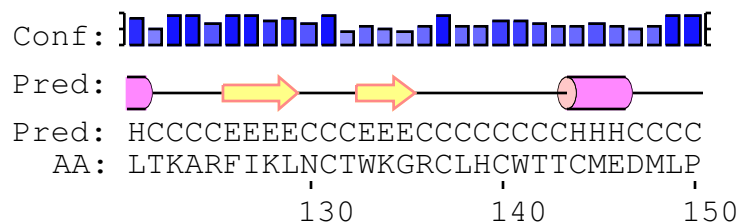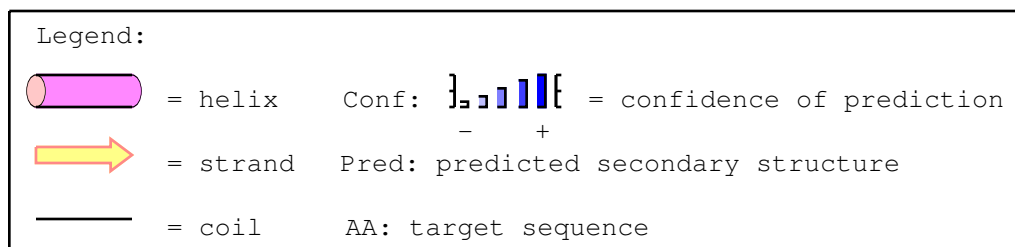

Supplement: Supplementary file 3 — Additional file 3: HPV6 E6 variation sequence predicted secondary structure. Secondary structure within the variation sequence of HPV6 E6 protein. Variations of HPV6E601, HPV6E602 and HPV6E603 were integrated into one sequence to predict. (PDF 15 kb) [file 12985_2019_1221_MOESM3_ESM.pdf]

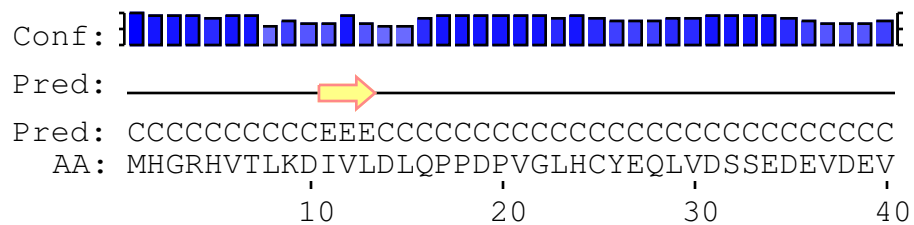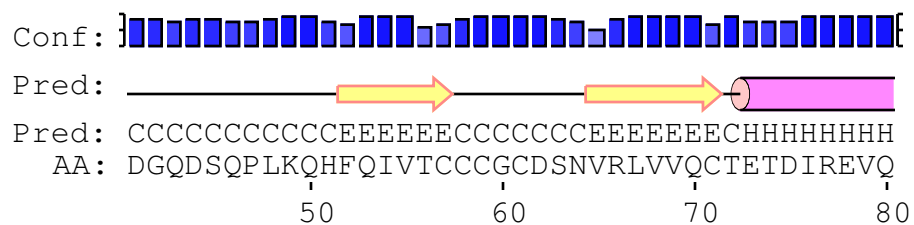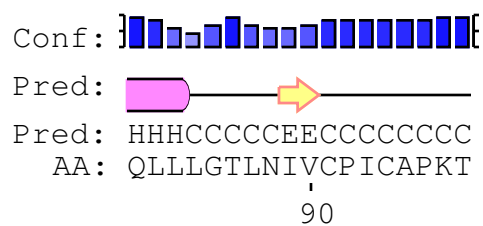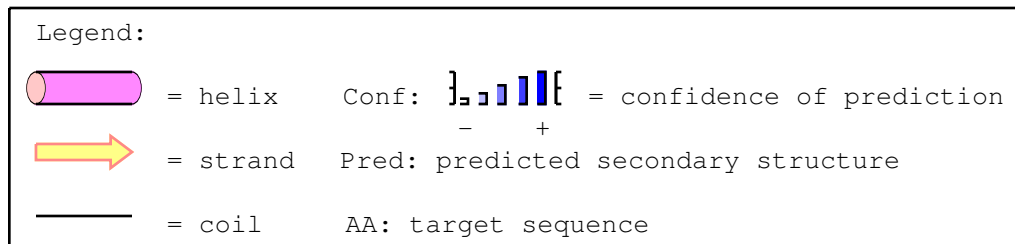

Supplement: Supplementary file 4 — Additional file 4: HPV6 E7 reference sequence predicted secondary structure. Secondary structure within the reference sequence of HPV6 E7 protein. (PDF 11 kb) [file 12985_2019_1221_MOESM4_ESM.pdf]

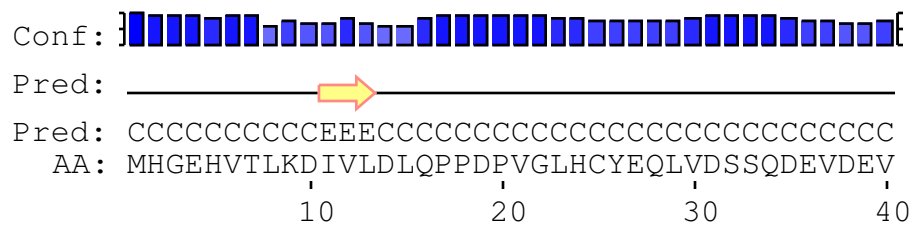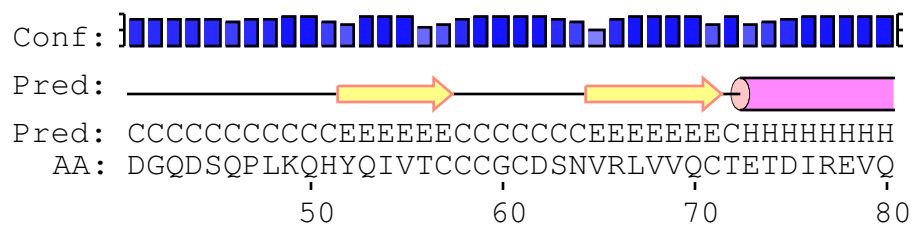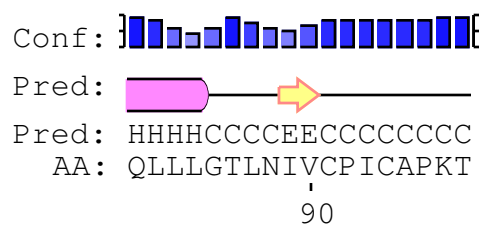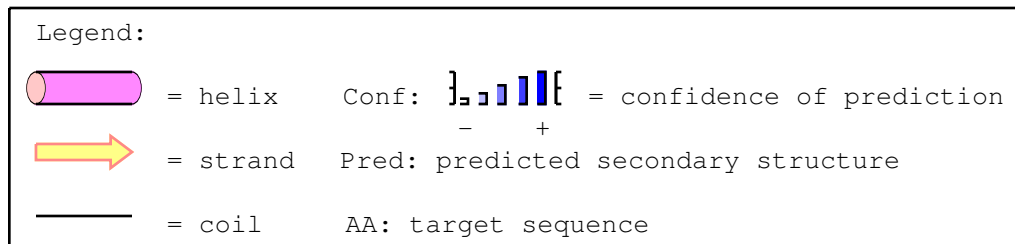

Supplement: Supplementary file 5 — Additional file 5: HPV6 E7 variation sequence predicted secondary structure. Secondary structure within the variation sequence of HPV6 E7 protein. Variations of HPV6E701, HPV6E702 and HPV6E703 were integrated into one sequence to predict. (PDF 11 kb) [file 12985_2019_1221_MOESM5_ESM.pdf]
